# Diabetes, Polycystic Ovarian Syndrome, Obstructive Sleep Apnea, and Obesity: A Systematic Review and Important Emerging Themes

**DOI:** 10.7759/cureus.26325

**Published:** 2022-06-25

**Authors:** Zarna Bambhroliya, Joel Sandrugu, Michael Lowe, Oluwasemilore Okunlola, Shafaat Raza, Stephen Osasan, Sudiksha Sethia, Tayyaba Batool, Pousette Hamid

**Affiliations:** 1 Research, California Institute of Behavioral Neurosciences & Psychology, Fairfield, USA; 2 Neurology, California Institute of Behavioral Neurosciences & Psychology, Fairfield, USA

**Keywords:** visceral adiposity, metabolic syndrome, diabetes type 2, metabolic dysfunction, obesity, hyperandrogenism, polycystic ovary syndrome, obstructive sleep apnea, insulin resistance

## Abstract

Type 2 diabetes mellitus (DM), polycystic ovarian syndrome (PCOS), obstructive sleep apnea (OSA), and obesity represent four large and growing patient populations. A great deal of scientific and clinical knowledge has been developed for them individually, and significant advancements made. Taken as a group, however, the interrelationships are not as well understood. The purpose of this systematic review is to identify the body of existing research that ties them together and then to identify and discuss the prevailing themes, particularly for cause-and-effect mechanisms. PubMed, Google Scholar, and ScienceDirect were used to identify systematic reviews and meta-analysis articles to establish the broadest reach. Initially, 434 articles were carefully screened, out of which 22 most relevant studies were reviewed. Five important themes were distilled from these papers based on continued and consistent emphasis in the literature. These themes include topics such as the importance of considering visceral obesity rather than Body Mass Index (BMI), the most effective treatment approaches, including mounting support for melatonin and circadian rhythm management, the results of OSA in its feed-forward contribution to hormone imbalance, the role of non-obesity-related risk factors to PCOS and OSA such as age and genetic predisposition, and growing evidence to suggest the importance of mental health as a comorbidity in addition to the more traditional ones such as cardiovascular pathology. A new framework for investigating the interaction across these four disorders is offered that includes a revised perspective on the specific role of PCOS, perhaps being further upstream relative to the others. There currently exists a lack of well-designed randomized controlled trials in this particular area of medicine, an endeavor we believe could result in significant value, particularly as it relates to treatment approaches.

## Introduction and background

Consider the following prevalence data: Diabetes mellitus (DM) remains a “giant” at 463 million people, or 9.3% of the population, projecting to 578 million by 2030 [1]. Polycystic ovarian syndrome (PCOS) has been reported in 21% of women of reproductive age worldwide [2]. Forty percent of women who are 18 and older are overweight, with 15% obese (BMI > 30) [3]. Nine hundred and thirty-six million adults globally experience mild obstructive sleep apnea (OSA), and 425 million show severe symptomology [4]. 

Martins and Conde explored the shared mechanisms connecting OSA and metabolic derangements, but their review focused on gender differences and not on the PCOS population [5]. Li et al. explored bariatric surgery for women with PCOS in a 2019 meta-analysis [6]. Findings were encouraging: surgery not only reduce BMI but also abnormal menstruation and DM prevalence. However, it reviewed a specific interventional approach. Missing is a broad assessment of reviews specifically investigating causal linkages and mechanisms amongst DM, PCOS, obesity, and OSA.

PCOS is an endocrine disorder, with androgens, insulin, and progesterone outside of normal ranges. Small cysts can be observed on the outer edges of the enlarged ovaries. Symptoms include menstrual irregularity, hair loss, and acne. Obesity is frequently observed. OSA results from upper airway blockage during sleep, and features snoring and daytime sleepiness. Obesity is at the top of the list of causes. Swollen tonsils, cardiovascular dysfunction, and endocrine disorders have also been implicated [7]. 

Is obesity a cause or effect of PCOS and OSA? We assess current evidence from review articles, identify established cause-and-effect relationships, and summarize findings of interest to endocrinologists, OB-GYNs, any healthcare provider concerned with BMI management, and even dentists, who play an increasing role in the screening for and treatment of OSA [8]. 

## Review

Methods

Two co-authors independently conducted article search and retrieval. The date of the last search was March 23, 2022.

*PubMed* Keywords: insulin resistance, obstructive sleep apnea, polycystic ovary syndrome Filters: “All Fields,” with no historical timeline filter

*Google Scholar* Keywords: polycystic ovary syndrome, obstructive sleep apnea, hyperandrogenism, obesity, insulin resistance Filters: Since 2018 (five years), and Type = Review Articles

*ScienceDirect* Keywords: insulin resistance, metabolic dysfunction, obstructive sleep apnea, polycystic ovary syndrome Filters: No historical timeline filters, and Type = Review/Research Articles 

Apart from the five years time frame filter for Google Scholar, and type filter of review/research articles for Science Direct, no other criteria were used, including peer review, publication status, setting (hospital vs. out-patient), location, original publication language, or any population attributes. Additionally, keywords were chosen (“insulin resistance” instead of “diabetes”) to avoid dilution by extremely broad topic headings. Each article was qualified based on relevance to the topic (author discretion) - initially at the title and abstract level and subsequently upon detailed review. The decision was made post-review to limit the assessment of remaining papers to only systematic reviews and meta-analysis publications. All qualified articles were available in full-text format. Critical appraisal was independently performed using the scale for the assessment of narrative review articles (SANRA) tool by two co-authors to grade the aggregate level of quality of the literature being reviewed [9]. This systematic review was done according to the PRISMA guidelines.

Results

**Figure 1:**
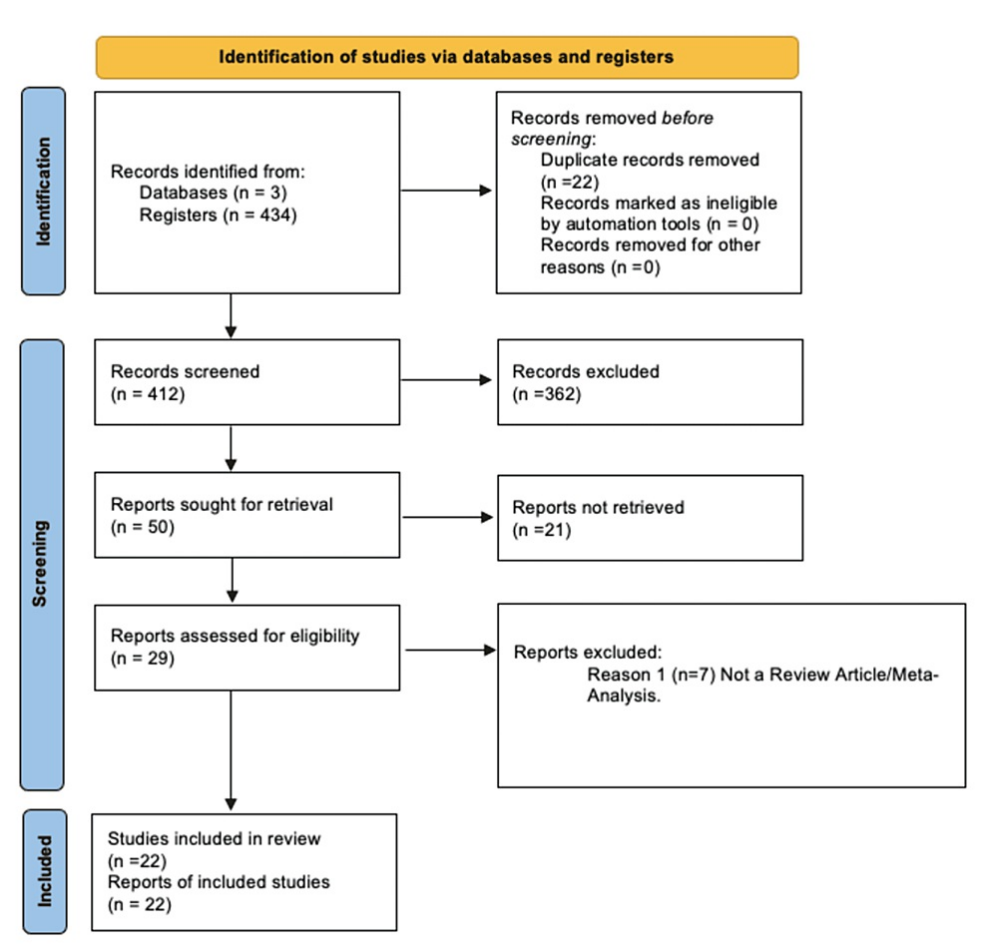
Prisma Flow Diagram PRISMA: Preferred Reporting Items for Systematic Reviews and Meta-Analyses

Four hundred and thirty-four studies were initially returned (Figure 1). Twenty-two duplicates were excluded, and 412 studies were screened from the abstracts. Another 362 articles were excluded based on lack of relevancy at the abstract level. The remaining 50 studies were reviewed in detail for relevancy; 21 were disqualified. Of the 29 remaining papers, seven were discarded as a result of not being review articles.

A summary of the conclusions of our qualified studies can be found in Tables 1, 2.

**Table 1:** Summary of Conclusions of Selected Articles OSA: obstructive sleep apnea, PCOS: polycystic ovary syndrome, CPAP: continuous positive airway pressure, IGT: impaired glucose tolerance, COCP: combined oral contraceptives, IR: insulin resistance, T2DM: type 2 diabetes mellitus, WAT: white adipose tissue, VAT: visceral adipose tissue

Author	Year	Publication Type	Stated Relevant Conclusions
Kahal, et al. [10]	2018	Meta-analysis	OSA is associated with obesity and worse metabolic profiles in women with PCOS. However, whether the effects of OSA are independent of obesity remains unclear.
Shah [11]	2019	Literature Review	PCOS is a common and complex condition with multi-faceted etiologic origins and represents a major long-term health risk for affected adolescents and women.
Dwivedi, et al. [12]	2020	Literature Review	The pathogenesis of PCOS still remains unclear.
Carneiro, et al. [13]	2018	Literature Review	Weight control is essential to decrease the risk of cardiovascular events and mortality potentially linked to both obesity and OSA. CPAP seems to treat only OSA without decreasing these risks.
Ortiz-Flores, et al. [14]	2019	Literature Review	PCOS is the most frequent endocrine disorder in premenopausal women. Treatment must be chronic and personalized, adapted to the needs of each patient throughout their life, aimed at improving the symptoms of androgen excess and ovulatory dysfunction, and to prevent, or even treat, early associated metabolic complications.
Tasali, et al. [15]	2008	Literature Review	Affected women (of PCOS) are at increased risk for the development of early-onset IGT and type 2 diabetes mellitus.
Vgontzas, et al. [16]	2003	Literature Review	Accumulating evidence provides support to our model of the bi-directional, feedforward, pernicious association between sleep apnea, sleepiness, inflammation, and insulin resistance, all promoting atherosclerosis and cardiovascular disease.
Neven [17]	2018	Literature Review	Clinicians should focus on lifestyle adjustments as the first-line management to improve reproductive, metabolic, cardiovascular, and psychosocial outcomes focusing on weight management and physical exercise. In addition, pharmacological therapy in the form of COCPs and metformin may be useful.
Witchel, et al. [18]	2020	Literature Review	Healthy lifestyle interventions with the prevention of excess weight gain comprise the primary intervention for all comorbidities. Hence, early identification of girls “at risk” for PCOS and those with PCOS is a priority.
Sam, et al. [19]	2021	Literature Review	Current evidence from clinical and population-based studies indicates that OSA occurs at increasing frequency among women with PCOS. Age and obesity appear to be major determinants in this association. In addition, observational evidence in a limited number of studies suggests that metabolic outcomes are worsened among PCOS women who have OSA.
Ehrmann, et al. [20]	2012	Literature Review	The risk for OSA is at least 5- to 10-fold higher compared to the risk in similarly obese women without PCOS.
Ip, et al. [21]	2007	Literature Review	Apart from the common risk factor of obesity, increasing data also support that OSA exerts independent adverse effects on glucose intolerance/diabetes mellitus, although definitive evidence is still needed.
Schmiedt, et al. [22]	2021	Literature Review	IR is a heterogeneous state, which plays an important role in the pathogenesis of metabolic syndrome, T2DM, and several endocrine diseases.
Barber, et al. [23]	2019	Literature Review	It is widely accepted based on current evidence that weight gain and obesity are important risk factors for the clinical and biochemical manifestations of PCOS in those women who are genetically predisposed.
Helvaci, et al. [24]	2021	Literature Review	There is good evidence indicating that women with PCOS have an increased prevalence of cardiometabolic risk factors, prediabetes, and diabetes and are at risk for the development of OSA, endometrial cancer, and mood disorders during their early reproductive years.
Celik, et al. [25]	2021	Literature Review	Although these cardiometabolic risk factors are more common among women with PCOS, currently there is no strong evidence for increased cardiovascular morbidity and mortality in aging women with PCOS.
Kahal, et al. [26]	2020	Meta-analysis	35.0% (95% CI 22.2%-48.9%) of women with PCOS had OSA. OSA prevalence was markedly higher in obese versus lean women with PCOS, and in women with PCOS compared to controls (odds ratio = 3.83, 95% CI 1.43–10.24, eight studies, 957 participants (349 PCOS and 608 controls).
Piri-Gharaghie [27]	2021	Literature Review	PCOS can be considered a complex, heterogeneous metabolic syndrome triggered or maintained by the combined effect of inheritable genetic susceptibilities and environmental risk factors.
Livadas, et al. [28]	2022	Literature Review	The association of PCOS with increased T2D risk is relatively robust and thus should not be neglected in any woman with the syndrome.
Spinedi, et al. [29]	2018	Literature Review	Many metabolic-reproductive alterations associated with PCOS are closely dependent on WAT dysfunction, particularly at the VAT pad level. However, the increase in VAT pad mass per se is not an unequivocal indication of VAT dysfunction, whereas the development of enlarged local adipocytes is indeed a key factor.
Sam, et al. [30]	2019	Literature Review	Sleep disturbances are common in PCOS, although most studies so far are limited by a small sample size and have been conducted in clinic-based cohorts with referral bias and overrepresentation of women with more severe symptoms.
Bahman, Hajimehdipoor, et al. [31]	2018	Literature Review	Thus, sleep modification can be effective in neurohormonal regulation and management of PCOS.

**Table 2:** Summary of Publication Source and Research Location

Author	Year	Journal	Country
Kahal, et al. [10]	2018	Sleep Research Society	United kingdom
Shah [11]	2019	Children	USA
Dwivedi, et al. [12]	2020	Journal of Clinical Trials	China
Carneiro, et al. [13]	2018	Metabolism	Brazil
Ortiz-Flores, et al. [14]	2019	Medicina Clinica	Spain
Tasali, et al. [15]	2008	Sleep Medicine Clinics	China
Vgontzas, et al. [16]	2003	Journal of Internal Medicine	USA
Neven [17]	2018	Seminars in Repro Medicine	Netherlands
Witchel, et al. [18]	2020	Pediatric Research	USA
Sam, et al. [19]	2021	Endocrine and metabolic Research	USA
Ehrmann, et al. [20]	2011	Steroids	USA
Ip, et al. [21]	2007	Sleep Medicine Clinics	China
Schmiedt, et al. [22]	2021	Acta Marisiensis	Romania
Barber, et al. [23]	2019	SAGE	United Kingdom
Helvaci, et al. [24]	2021	Wiley	Turkey
Celik, et al. [25]	2021	J Turk Ger Gynecol Assoc.	Turkey
Kahal, et al. [26]	2020	Sleep and Breathing	United kingdom
Piri-Gharaghie [27]	2021	Personized Medicine Journal	Iran
Livadas, et al. [28]	2022	World Journal of Diabetes	Greece
Spinedi, et al. [29]	2018	Int Journal of endocrinology	Argentina
Sam, et al. [30]	2019	SAGE	USA
Bahman, Hajimehdipoor, et al. [31]	2018	Int Journal of Preventive Medicine	Iran

It can be seen in Table 2 that the largest number of studies, with six total, was published out of the United States. China and the United Kingdom each had three, followed by Turkey and Iran with two each. In total, 10 different countries were represented.

Discussion

Several key findings were identified in our review.

Mental Health May Be an Important Confounding Variable and Comorbidity

Kahal et al. (2018) offer a search of eight databases with no filters, yielding six studies and 252 patients [10]. The authors stated that the relationship between OSA and obesity in women with PCOS is unclear. In terms of future research direction, fertility, mental health, cardiovascular health, and risk for Type 2 DM were highlighted. Mental health is interesting as it has been proposed as a key measure for assessing outcomes. Dokras et al. evaluated women with PCOS using the Short Form-36 tool, which includes attributes such as social functioning and mental health. All six studies exhibited a risk of bias for confounding variables. The paper points out that both OSA and PCOS independently are correlated with depressed mood, an area identified for targeted research [32-33]. The fact that having poor quality sleep would lead to poor mood comes as no surprise. The confounding role of PCOS is less understood [34].

Shah’s review covers co-morbidities, with themes including cardiovascular disease and, again, mood disorders [11]. For patients specifically co-diagnosed with mental health disorders, one of the studies reviewed demonstrated that mindfulness programs vs. placebo control resulted in not only improved mental health measures but also physiological reduction of salivary cortisol levels [35]. Dwivedi et al. additionally summarized comorbidities [12]. Noted were cardiovascular disease, OSA, fertility and ovulation, and what the authors referred to as, “psychosocial elements.” In terms of lifestyle vs. biopharmaceutical interventions, they suggest that drugs should constitute the primary treatment plan. Oral contraceptives are mentioned for increased androgen levels and anovulation; citrate is mentioned for infertility. Finally, it is noted that PCOS patients should be screened for OSA in a clinical setting. 

OSA May Be Exerting a Feed-Forward Impact in the Form of Hormone Imbalances

Carneiro et al. specifically reviewed cardiovascular disease as a comorbidity with obesity and OSA [13]. They explored OSA-related hormones that act on vasoconstrictor system activation (angiotensin II and endothelin), aldosterone, hypothalamic-pituitary-adrenal (HPA) axis products (such as cortisol), IGF-1, sex hormones, and even leptin [36-42]. They concluded that angiotensin II may actually contribute to renal disease. Here, it stimulates a highly potent vasogenic cytokine (vs. acting as a pressor agent) [43]. Given the causal effect of DM on renal dysfunction, it is easy to see how an OSA-driven renal pathology is an area for further research. Their hypothesis is drawn from animal studies in which losartan (angiotensin receptor blocker) did not preempt blood pressure rise in rats with hypoxia [44]. OSA as a source of hormonal imbalances, as opposed to a result of them, would lend support to positioning it earlier in the cause-and-effect flow stack than previously thought. 

Distribution of Body Fat (Visceral Adiposity) Is Likely More Important Than Raw BMI in Considering a Patient’s Weight 

The Carneiro review also posits that “obesity is not just obesity.” BMI is not an appropriate measure they argue, at least in terms of predictive value for OSA. This view holds support from other literature [45-46]. OSA prevalence has been observed at increased levels in patients with increased waist circumference, waist-to-hip ratio, and neck circumference [47-48]. Ortiz-Flores et al. argued that diet should be the first-line treatment [14]. The key aims acknowledge the sub-classification of obesity. They include achieving redistribution of body fat, curtailing excessive androgen levels, and improving insulin resistance. Escobar-Morreale et al. also proposed surgery as an option and noted improved outcomes across reproductive parameters, androgen levels, insulin sensitivity, and resolution of ovulatory dysfunction in patients (BMI > 35) post-surgery [49].

Tasali et al. looked at the link between OSA and PCOS [15]. They focused on “visceral adiposity” and argued that elevated BMI is not the right measure. The mechanism offered is that visceral fat is more active from a metabolic standpoint. Other researchers have found that visceral fat specifically is more highly correlated with OSA [50]. They speculated that males show a higher prevalence of OSA than females because the ratio of visceral fat as a percentage of total body fat is higher in men. Visceral obesity is further explored by Vgontzas et al. [16]. They reinforced that sleep apnea can be a causal factor in PCOS, regardless of obesity. They proposed a bi-directional model and feed-forward between OSA and insulin resistance. They were clear that both visceral obesity and insulin resistance are multi-factorial, owing to genetic and environmental risk factors. Taken together, visceral obesity and insulin insensitivity lead to sleep apnea. It is further suggested that the resulting sleep apnea can accelerate metabolic derangements. They argued that treatment should focus on diet, exercise, and sleep as opposed to pharmaceuticals. 

Neven et al. provided a summary of the diagnosis and treatment of PCOS [17]. It reviewed the Rotterdam diagnostic criteria, which require any two of oligo-ovulation, anovulation, clinical hyperandrogenism, biochemical hyperandrogenism, or polycystic ovaries [51]. They call for lifestyle modification as the first-line treatment approach, suggesting that it improves reproductive, metabolic, psychological, and cardiovascular comorbidities. Witchel et al. suggested poor clinical PCOS diagnosis performance, specifically blaming unclear criteria and physician knowledge gaps [18]. They offer a de novo set of diagnostic criteria and emphasize the importance of early identification of “at risk” patients. They suggest that a PCOS diagnosis should also prompt the evaluation of applicable family members, reinforcing both the genetic component and the effectiveness of early intervention via lifestyle modification. An interesting point is made that successful treatment of OSA actually has no impact on DM and that the forward-looking focus of OSA in PCOS patients should be the management of symptoms. Obesity alone does not account for the OSA prevalence in women with PCOS. 

Sam and Tasali looked specifically at the role that OSA may play in metabolic disorders within the PCOS patient population [19]. Contrary to the lack of a clear association, as suggested by Kahal et al. (2018), these authors point to the increased prevalence of OSA occurring later in life, a point based on smaller studies. The larger studies they assessed showed that the OSA-PCOS link is not completely accounted for by obesity [52]. Ehrmann’s review looked at aspects of the OSA-PCOS connection, including sex differences related to epidemiology, sex steroids, body fat distribution, and comorbidity of PCOS with cardiovascular disease [20]. There is an agreement with the conclusions from Sam and Tasali - obesity alone cannot fully account for the prevalence of OSA with PCOS. In one of the papers reviewed, Fogel et al. demonstrated that PCOS patients were more likely to have disordered breathing while sleeping and daytime sleepiness, even when controlling for BMI [53]. 

Ip et al. looked at the link between OSA and DM [21]. They pointed out that, at a minimum, OSA exerts independent (from obesity) effects. Schmiedt et al. looked from the insulin resistance angle and assessed mechanisms for insulin increasing steroid levels in women with PCOS [22]. An important contribution was its emphasis on how steroid levels are increased. Sex hormone-binding globulin levels are reduced with increased insulin. Increased androgens levels result from the direct stimulation of thecal cell secretion by insulin. This elevation occurs as a result of increasing two enzymes, in particular, CYP17A1 and p450scc [54-56]. Barber et al. focused on mechanisms of pathology and treatment approaches. Mechanisms trinucleotide repeats, as well as second messenger signaling pathways [23]. Mohlig et al. suggested that the number of CAG repeats within the androgen receptor gene directly contributes to insulin resistance in patients with PCOS [57]. Again, there is no direct tie to obesity, per se. Barber et al. have shown that the phosphatidylinositol 3-kinase pathway, which mediates the effect of glucose uptake into skeletal muscle, features a post-receptor defect [58]. They state that the PCOS population includes obese women who have developed PCOS for other reasons.

Helvaci and Yeldiz reviewed aging and menopause as a correlating to PCOS [24]. They suggested the risk of cardiovascular disease is no difference between women with and without PCOS. They mentioned challenges with following generational cohorts. They are also skeptical about interpreting a direct linkage between PCOS and OSA. Limitations are pointed out: cross-sectional design, small sample sizes, selection bias, range and variability of BMI values, and ongoing confounding factors. The meta-analysis referencing the eight studies with these flaws found a 32% prevalence (95% CI of 13%-55%) of OSA in PCOS women [59]. They conclude that “it is difficult to determine a causal link between PCOS and OSA based on these reports.” Celik et al. elaborated on the theme of PCOS in women of advanced age [25]. The literature reviewed was mixed on whether or not PCOS in these women persists vs. healthy control counterparts. Although confirmation is given for the classic PCOS comorbidities, including obesity, insulin resistance, DM, hypertension, and cardiovascular disease, the causal linkage - at least in older women - is left as an unanswered question.

Another paper from Kahal investigated the prevalence of OSA in women with PCOS [26]. Seventeen studies (n=648 in aggregate) were selected. Thirty-five percent of PCOS women had OSA (95% CI of 22.2%-48.9%). Helvaci et al. concluded that 32% of these two data points support one another. The Kahal (2020) review discovered that OSA prevalence was higher in obese women, including an Odds Ratio of 3.83 (95% CI of 1.43-10.24). This sub-analysis was limited to eight studies and 957 women. Piri-Gharaghie et al. focused on genetic factors as a contributor to PCOS [27]. A summary of the known PCOS genes was provided (CYP11A1, CYP17, androgen receptor, sex hormone-binding globulin (SHBG), the insulin gene, and the insulin receptor gene). They remain non-committal on lifestyle and controllable risk factor modification vs. drugs. It is suggested based on available evidence that while lifestyle modifications can improve PCOS-associated abnormalities, environmental insults such as smoking, pollution, and dietary toxins unmask the genetic predisposition [60-61].

Livadas et al. describe a “controversial association” between PCOS and diabetes [28]. Menopausal women are identified as being at risk for PCOS. This positioning of a “can’t miss” diagnosis is clinically relevant. They also favor lifestyle modification over pharmacological intervention. While there is some argument that obesity is a contributing factor to OSA in women with PCOS, there is also little doubt that it is not the sole contributor. Evidence also points to aging and genetic predisposition based on mechanisms ranging from trinucleotide repeats to defective receptors.

Circadian Rhythms and Melatonin Represent Viable Treatment Approaches

Spinedi et al. reviewed melatonin as a therapy. Dysfunction of the melatonin receptor has been linked to an increased risk of developing PCOS [29]. In contrast to the “diet first” approach supported by Ortiz-Flores et al. these authors favor insulin sensitizers and secretagogues. Sam and Ehrmann (2019) summarized links between sleep disturbances and PCOS [30]. They touch on melatonin, establishing support for Spinedi. The study cited, however, was only n=26 [62]. Melatonin had previously been proposed by Li et al. SNPs in the melatonin receptor gene were associated with PCOS in a Genome-Wide Association Study of n=500 women [63]. Hajimehdipoor et al. emphasized that sleep hygiene should be included with diet and exercise [31]. Obesity and even insulin resistance are the result of low-quality sleep and have been demonstrated to contribute to PCOS [64-65].

A New, Revised Framework

**Figure 2:**
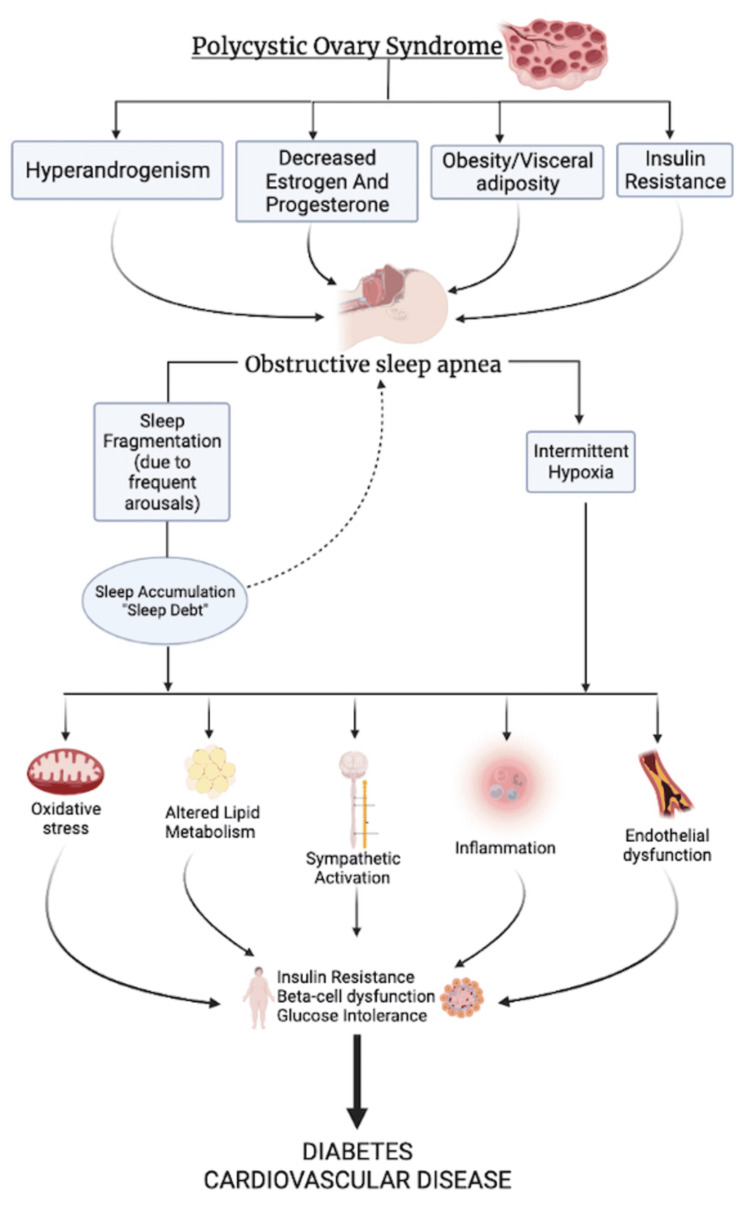
Summary Framework Schematic illustration of Interrelating connection between polycystic ovary syndrome, obstructive sleep apnea, insulin resistance and obesity. (This image was created with Biorender.com)

We offer a new, slightly revised framework, depicted in Figure 2. PCOS, the least diagnosed of the conditions, is the starting point. It represents a diagnostic call-to-action that builds awareness around the most targeted patient profile, females of reproductive age.

Although PCOS and OSA both feature awareness campaigns in the market, OSA benefits from having an entire dental sleep medicine academy focused on it [66]. Several feed-forward causal effects flow downstream, including hormone imbalances. Obesity is labeled as visceral adiposity and not BMI. Insulin resistance in our model is attributed to PCOS, not the other way around. The resulting derangements manifest as OSA. As sleep deficit accumulates, there is an alteration in lipid metabolism and an increase in the sympathetic drive. OSA can include up to 30 discrete breathing interruptions of 10 seconds or greater per hour [67]. The alteration in lipid metabolism as the body fights to breathe, in what is essentially a “fight or flight” response, eventually leads to beta-cell dysfunction and glucose intolerance. The final common pathway is DM. This framework also allows a forward flow from PCOS to DM that does not depend on obesity. The diabetic state feeds back to several points earlier before it, most notably insulin resistance, which supports the PCOS-to-OSA flow path.

## Conclusions

It is paramount to recognize that while BMI is a popularized measurement tool, it offers little value in assessing PCOS, OSA, and DM interrelationships. Numerous papers offered alternative methods such as neck circumference and waist-to-hip ratio. Treatment plans may be able to better target redistribution of fat, as opposed to reduction of BMI. Treatment overall remains controversial, with most research favoring non-pharmacological management as first-line. Melatonin and circadian rhythm management may play a role in patient care. Surgery has also been proposed as an intervention.

OSA can be prevalent in the PCOS population without obesity. And even in obese PCOS patients presenting with OSA, obesity is likely not the exclusive causal factor. Factors include advancing age and genetic predisposition for insulin resistance. We have offered a slightly modified framework to describe their interrelationship. PCOS is introduced as the most upstream diagnosis, exerting a forward effect that culminates most directly as OSA. Both of these pathologies taken together exert a causal effect on insulin resistance and ultimately, DM. Then, DM closes a feedback loop back up across all predisposing factors. Obesity in these patients can initially result directly from the PCOS. Finally, this literature highlights the importance of mental health comorbidities. In addition to others like cardiovascular diseases, they may represent an important consideration to the patient management continuum, from diagnosis to measurement of outcomes. RCTs analyzing benefits of drug-based vs. lifestyle modification treatments in obese patients with PCOS, DM, and OSA, would be of value to the scientific and clinical communities.
